# Severe Pulmonary Arterial Hypertension: Comprehensive Evaluation by Magnetic Resonance Imaging

**DOI:** 10.1155/2015/946920

**Published:** 2015-09-08

**Authors:** El-Sayed H. Ibrahim, Abubakr A. Bajwa

**Affiliations:** ^1^University of Michigan, Ann Arbor, MI 48105, USA; ^2^University of Florida, Jacksonville, FL 32209, USA

## Abstract

Pulmonary arterial hypertension (PAH) is characterized by elevated pulmonary artery (PA) pressure, which negatively affects the right ventricular (RV) function. This report shows a patient with severe PAH, on whom a comprehensive MRI exam was performed to evaluate both PA and RV. New imaging sequences were implemented for obtaining additional parameters about the patient's condition. The results show the capabilities of the developed exam of providing complete picture of the cardiovascular system in PAH, which helps the physician optimize treatment.

## 1. Introduction

Pulmonary arterial hypertension (PAH) is characterized by an elevated pulmonary artery pressure (PAP), which increases the right ventricular (RV) afterload. Although PAP measurement by right heart catheterization (RHC) is the gold standard for confirming PAH, the technique's invasiveness and association with small, yet serious, risks of morbidity and mortality preclude its use on a regular basis. Furthermore, it is actually the RV condition that predicts the patient's survival in PAH [1]. Therefore, both the pulmonary artery (PA) compliance and RV function should be studied as one unit for a thorough assessment of PAH, especially in advanced disease stages. Magnetic resonance imaging (MRI) has been recently considered in PAH [2, 3], where different criteria have been evaluated for estimating PAP [4]. Furthermore, different MRI techniques have been developed for measuring parameters representing PA hemodynamics [5], PA vessel wall characteristics [6], RV function [7], and left ventricular (LV) involvement [8]. However, there is no report about the combined use of these techniques for a comprehensive evaluation of PAH. In this case, we show the implementation of advanced MRI techniques for evaluating both the RV function and PA compliance in severe PAH for better understanding of the patient's condition. Standard images from X-ray, echocardiography, and Doppler are also shown. To the best of our knowledge, this report is the first to illustrate the importance and capabilities of MRI for providing a large group of cardiovascular parameters that complement each other for a thorough assessment of PAH and better treatment planning.

## 2. Case Presentation

**Figure 1:**
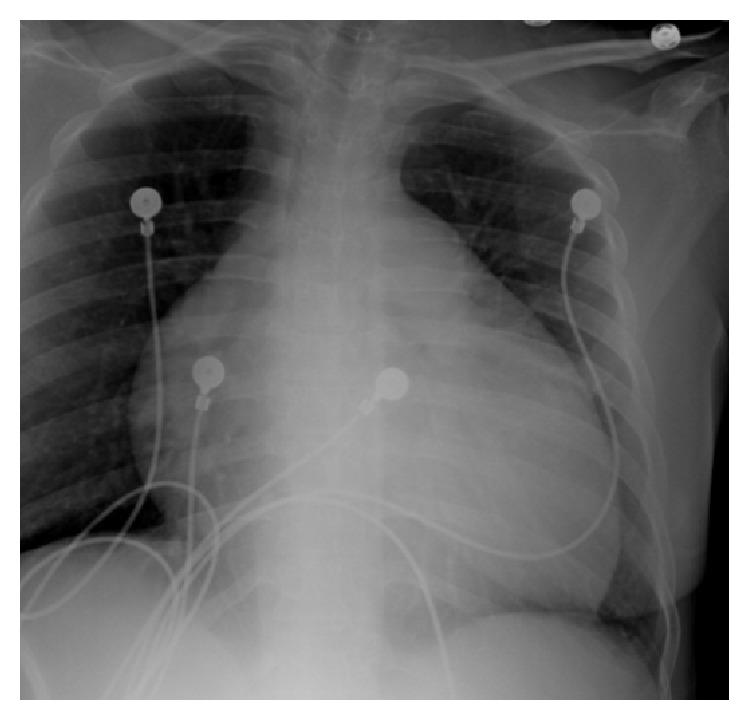
Chest X-ray image showing a significantly enlarged cardiac silhouette.

**Figure 2:**
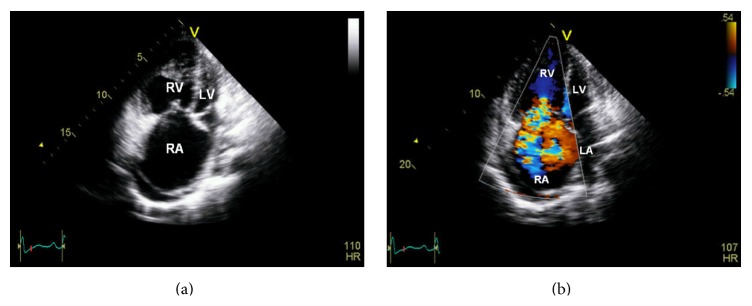
(a) Echocardiography apical view at end systole showing markedly enlarged right ventricle (RV) and right atrium (RA) and compressed left ventricle (LV). (b) Color echo Doppler image showing severe tricuspid regurgitation. LA = left atrium.

A 28-year-old African-American female presented to our clinic with symptoms of syncope and dyspnea on exertion. RHC confirmed severe PAH with mean PAP of 100 mmHg. Standard 6-minute walking distance (6MWD) and probrain natriuretic peptide (proBNP) tests showed deteriorated heart condition: 348 m and 2566 pg/mL, respectively. Chest X-ray showed significantly enlarged cardiac silhouette (Figure 1). Echocardiography showed markedly dilated RV and right atrium (RA) and compressed LV (Figure 2(a)). Echo Doppler showed severe tricuspid regurgitation (Figure 2(b)).

A comprehensive MRI exam was ordered to fully investigate the cardiovascular system condition and quantify various relevant parameters. The study was approved by the institutional review board and the patient provided an informed consent. The MRI exam was performed on a 3.0 Tesla Siemens MAGNETOM Trio MRI scanner (Siemens Medical Solutions, Erlangen, Germany) using electrocardiogram gating, which lasted for 30 minutes. It included the following imaging sequences: (1) cine imaging to evaluate global heart function (ejection fraction (EF), ventricular volume index (VVI) = RV end-diastolic volume divided by LV end-diastolic volume, ventricular mass index (VMI) = RV mass divided by LV mass, and RA size (area on a four-chamber view at end systole)) and assess the degree of myocardial trabeculation; (2) strain-encoding (SENC) imaging [9] to measure the RV longitudinal strain (represented as a color-coded strain map on a short-axis (SAX) view); (3) velocity-encoding flow imaging perpendicular to the main PA (MPA) to measure the MPA diameter (during diastole), PA distensibility (change in MPA cross-sectional area between end diastole and end systole), and PA pulse wave velocity (PWV) [10]; and (4) velocity-encoding flow imaging through the tricuspid valve to measure the tricuspid regurgitation velocity.

For measuring PWV, time-resolved (cine) velocity-encoded MRI images were acquired perpendicular to the MPA during early systole. During postprocessing, the MPA was semiautomatically segmented in the acquired timeframes. The MPA cross-sectional area and through-plane flow were automatically calculated from the resulting magnitude and phase-encoded images, respectively. Area was then plotted against flow, and PWV was calculated as the slope of the line fitted to flow-area data using the least square-error method, such that PWV represents the ratio between flow change and area change during early systole.

**Figure 3:**
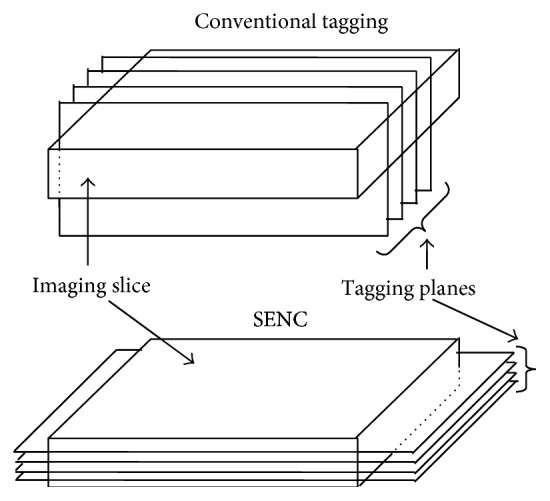
SENC imaging. SENC uses tagging as in conventional tagging. However, the tag planes are applied parallel to, and lie inside, the imaging slice; thus through-plane strain is measured in SENC.

Strain-encoding (SENC) is similar to conventional MRI tagging in that it is based on applying parallel planes of saturated magnetization that are used as virtual markers to track tissue motion. However, in contrast to conventional tagging where the saturated magnetization planes are applied* perpendicular* to the imaging plane and thus appear as dark stripes in the imaged slice, SENC applies the tagging planes* parallel to* (and therefore lie inside) the imaged slice, similar to pages of a book (Figure 3). Through-plane motion results in pushing these planes closer together during tissue contraction or further apart during tissue stretching, which results in increasing or decreasing the tagging frequency, respectively. Therefore, the SENC images do not show any dark stripes; rather SENC measures through-plane strain based on estimating the resulting tagging frequency. Therefore, longitudinal and circumferential strains are measured from SENC short-axis and long-axis images, respectively.

**Figure 4:**
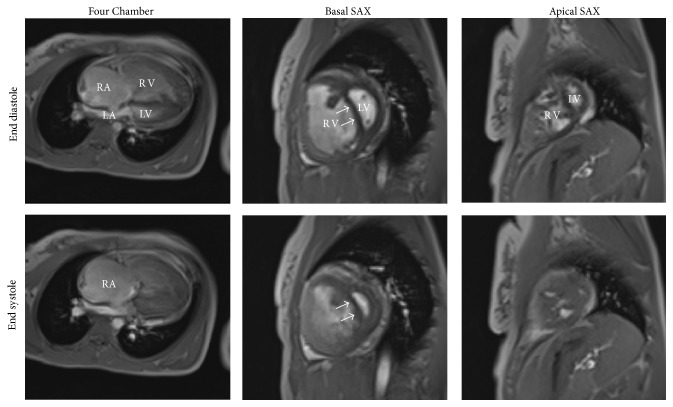
MRI images at end diastole and end systole. The images show markedly enlarged right ventricle (RV) and right article (RA) with leftward interventricular septal wall bowing (arrows). The RV is significantly trabeculated, especially at the apex. LV = left ventricle; LA = left atrium; SAX = short-axis.

**Figure 5:**
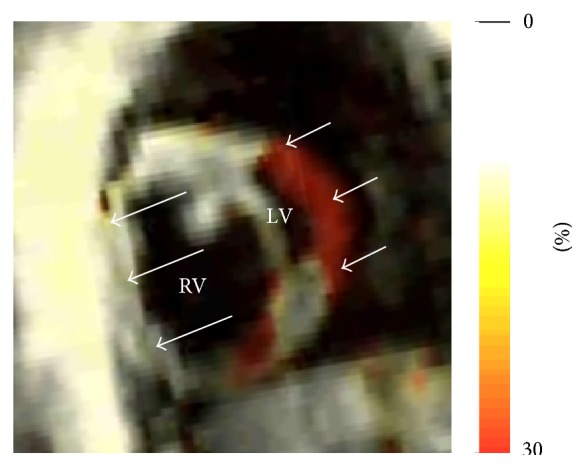
Short-axis strain-encoding (SENC) image showing color-coded longitudinal strain at end systole. Although the left ventricle (LV) is normally contracting (appears red, short arrows), the right ventricle (RV) shows negligible contraction (appears white, long arrows).

The cine MRI images (Figure 4) showed markedly dilated RV (VVI = 2.13) and RA (RA size = 9.6 cm^2^) with paradoxical leftward interventricular septal wall motion (arrows), especially during early diastole. As the LV mass is normally larger than RV mass, then VMI values <1 and >1 represent normal and pathological cases, respectively. In the presented case, VMI was 1.5, which reflects RV hypertrophy and trabeculations, as visually shown in the apical MRI slice in Figure 4. The results showed a deteriorated RV function (RVEF = 45%), albeit a normal LV function (LVEF = 80%). The RV myocardium was hypokinetic as evidenced by the SENC image in Figure 5, where RV peak longitudinal strain = 10.3%.

SENC images are typically acquired in a segmented fashion, which takes about 10 cardiac cycles of breath-holding to acquire images free from motion artifacts. However, we developed a non-Cartesian spiral acquisition of SENC, which allows for faster data acquisition to the extreme of collecting the whole k-space data in one heartbeat; that is, without breath-holding, although this comes at the cost of reduced image quality (e.g., smaller matrix or low spatial resolution). Actually, this development allowed for acquiring the SENC images in this patient who was not able to hold her breath. Nevertheless, even in the resulting low-resolution image, the noncontracting myocardium (appears white) could be easily distinguished from contracting myocardium (appears red) for better and accurate evaluation of regional heart function.

**Figure 6:**
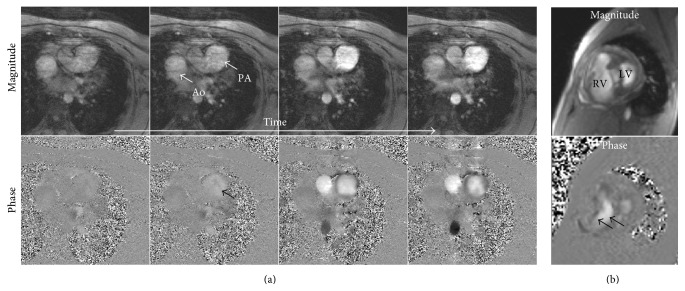
(a) MRI velocity-encoding magnitude (top) and phase (bottom) flow images through the main pulmonary artery at different time points during early systole. The images show a dilated pulmonary artery (PA), which appears larger than the aorta (Ao). The PA shows negligible change in cross-sectional area despite the flow increase with time (shown in the phase images). (b) MRI velocity-encoding magnitude (top) and phase (bottom) flow images through the tricuspid valve at end systole showing severe tricuspid regurgitation (arrows). LV = left ventricle; RV = right ventricle.

The flow images showed a significantly dilated PA (PA diameter = 3.5 cm), where the PA's cross section was larger than that of the aorta (Ao), as shown in Figure 6(a). The images showed negligible PA distensibility despite the noticeable flow increase with time (shown in the phase images). The relationship between flow and area of the PA resulted in elevated PWV (PWV = 3.33 m/s), representing a stiff arterial wall. Finally, the tricuspid flow images (Figure 6(b)) showed severe flow regurgitation (tricuspid regurgitation velocity = 4.45 m/s).

## 3. Discussion

This report illustrates the capabilities of the developed comprehensive MRI exam of evaluating severe PAH. The imaging protocol provides important parameters about both PA and RV conditions, which present a complete evaluation of the cardiovascular system in PAH. SENC is a relatively new imaging sequence that was included in the MRI exam for direct visual assessment of the RV myocardial contractility abnormalities. Longitudinal strain was measured because it is the main strain component in the RV. Despite the low resolution of SENC, the resulting image showed clear evidence of the compromised RV contractility compared to that of the LV. PWV is another important parameter that reflects arterial wall stiffness, a major component in PAH.

The presented case shows the importance of the developed MRI exam for providing important insights about PAH. PAH is characterized by dilated PA with increased vessel wall stiffness, which increases the RV afterload. The RV responds by building new sarcomeres in parallel (increased RV mass) and in series (increased RV volume). The undergoing ventricular remodeling compromises both the RV local and global functions, and it could lead to interventricular septal wall motion abnormality and heart failure in severe PAH, as in the present case. Other symptoms associated with PAH include increased tricuspid regurgitant flow, dilated RA, and limited physical capacity. The MRI exam in the presented case illustrates the various forms of the RV's response to the elevated afterload: remodeling through hypertrophy and dilatation, depressed local and global functions, abnormal interventricular septal wall motion, and right heart failure. Nevertheless, in the presented case, the LVEF was normal, most probably because of the disease etiology (classified by the World Health Organization (WHO) as group-1 pulmonary hypertension, which is not initiated because of left heart disease. A ventilation-perfusion (VQ) scan was performed on the patient with low probability, ruling out chronic thromboembolic pulmonary hypertension and other causes of PH). The results of the MRI exam help the physician optimize the treatment strategy for the patient. The patient in this report started on subcutaneous treprostinil and tracers medications, which makes her a good candidate for heart transplantation.

In conclusion, MRI has the capabilities of evaluating both the RV function and PA compliance. The developed protocol provides important measures of both entities in the same imaging session. The presented case shows an apparent coupling between the PA and RV conditions in severe PAH, which suggests that both entities should be studied together for comprehensive assessment of PAH, which would lead to better and individualized patient treatment.
